# Multiple birth rates of Korea and fetal/neonatal/infant mortality in multiple gestation

**DOI:** 10.1371/journal.pone.0202318

**Published:** 2018-08-15

**Authors:** Hyun Sun Ko, Jeong Ha Wie, Sae Kyung Choi, In Yang Park, Yong-Gyu Park, Jong Chul Shin

**Affiliations:** 1 Department of Obstetrics and Gynecology, College of Medicine, The Catholic University of Korea, Seoul, Republic of Korea; 2 Department of Biostatistics, College of Medicine, The Catholic University of Korea, Seoul, Republic of Korea; TNO, NETHERLANDS

## Abstract

**Objective:**

This study was conducted to analyze recent trends of multiple birth rates (MBR) and fetal/neonatal/infant mortalities according to the number of gestations in Korea.

**Methods:**

Data from 2009 to 2015 of live births, infant deaths and stillbirths were obtained from the Korean Vital Statistics. Neonatal mortality rate (NMR), infant mortality rate (IMR), and fetal mortality rate (FMR) in singleton, twin and triplet pregnancies were analyzed according to gestational period (GP; ≤ 23, 24–27, 28–31, and 32–36 weeks).

**Results:**

From 2009 to 2015, twin and triplet birth rates increased 34.5% and 154.3%, respectively. In twin births, NMR and FMR have been decreased significantly (from 10.92 to 8.62, p = 0.034 and from 41.00 to 30.55, p< 0.001, respectively), but IMR did not show significant decrease. There was no significant change of NMR, IMR, and FMR, in triplet births. Overall, in singleton, twin, and triplet births, NMR was 1.26 ± 0.09, 10.6 ± 1.12, and 34.32 ± 11.72, respectively, and IMR was 2.38 ± 0.26, 14.52 ± 1.38, and 41.13 ± 12.2, respectively. FMRs were 12 ± 1.73, 35.99 ± 3.55, and 88.85 ± 16.55, respectively, in singleton, twin, and triplet pregnancies. In spite of decreasing trends in overall mortalities, the odds ratios of NMRs and IMRs in 2015 were approximately 9-fold and 6-fold higher, respectively, in twin births, and approximately 37-fold and 20-fold higher, respectively, in triplet births, than those in singleton births. There were no significant differences in odds ratios of NMRs and IMRs at GP 32–36 among single, twin, and triplet births, although the odds ratios of FMR at GP 32–36 in triplet gestation was significantly higher than those in singleton and twin gestation.

**Conclusion:**

Neonatal/infant mortality in multiple births is still significantly high, which is mainly related with preterm birth. Close fetal monitoring is needed to prevent fetal death in triplet pregnancies, after 32 gestational weeks.

## Introduction

Advanced maternal age and the use of assisted reproductive technology (ART) procedures are considered as major factors associated with the increasing trend of multiple births [1–3]. Double and higher order embryo transfer is associated with a higher risk of stillbirth, neonatal/infant morbidity and mortality, when compared with single embryo transfer (SEF), mainly because multiple births are associated with preterm birth. Because preterm birth is not only related with neonatal/infant mortality and morbidity, but also predisposes to higher risks of chronic diseases and mortality later in life [4, 5], many countries are making efforts to reduce multiple birth rate (MBR), which have shown improved perinatal outcomes [6]. However, MBR in Korea has been increased steadily until 2008 and it was related with increased incidence of preterm birth. Among several public health indices, infant mortality rate (IMR) and neonatal mortality rate (NMR) are very important because the improvement of NMR and IMR directly influences the survival rate of the pediatric population. In addition, investigation of fetal mortality rates (FMR) during 2^nd^ and 3^rd^ trimester in multiple pregnancies can be valuable for planning antenatal care in multiple pregnancies, as well as for estimating benefits and risks related with a selective multifetal reduction in multiple pregnancies.

The aim of this study was to analyze the recent trends of multiple births and fetal/neonatal/infant mortalities among multiple gestations in Korea.

## Materials and methods

Data was obtained from the Korean Statistical Information Service [7]. Our study population included live births for the years 2009 through 2015, from the birth and fetal/infant death data files of the Statistics Korea (live births; n = 3,181,145, stillbirths; n = 43,385). We obtained approval from the Seoul St. Mary’s hospital institutional review boards (IRB) of Catholic University of Korea (KC17ZESI0265). IRB waived the need for consent, because records were accessed anonymously.

We excluded 5,142 infants and 2,166 stillbirths whose numbers of birth (singleton, twin, or triplet) were not stated, and 5 stillbirths whose number of birth was written as quadriplets. Thus, our final study population consisted of 3,176,003 live births and 41,214 stillbirths. Fetal death was defined as intrauterine fetal death occurring after 16 weeks’ gestational age and before the start of delivery or those occurring during labor. Neonatal death was defined as death occurring within the 28 days of life. Infant death was defined as death occurring within the first year of life. The MBR was defined as the ratio of multiple births per 1,000 live births. NMR was defined as number of deaths within 28 days per 1,000 live births. IMR was defined as number of deaths within 1 year per 1,000 live births. FMR was defined as number of fetal deaths within 1 year per 1,000 live births and fetal deaths. Gestational age is referred to by interval, in completed weeks; so, for example, a gestational age of 40 weeks means 40 weeks plus 0–6 days.

The data evaluated included: a) the annual number of total live births, singleton births, multiple births, twin births, and triplet births and MBR, twin births rate, triplet births rate, b) mean gestational age, births weight, proportion of preterm births, NMR, IMR, and FMRs in singleton, twin, and triplet births, c) the annual NMRs, IMRs, and FMRs in singleton, twin, and triplet births, c) the distribution of gestational age, in singleton, twin births and triplet births, d) NMRs and IMRs, stratified by 3 GPs (GP: 24–27, GP: 28–31, GP: ≥32 and < 37 weeks) and FMRs, stratified by 4 GPs (GP≤23, GP: 24–27, GP: 28–31, GP: ≥32 and < 37 weeks), in singleton, twin, and triplet preterm births.

All of the results were compared with the same parameters among the total live births during the same time period. Statistical calculations were performed with SPSS (version 24.0, Chicago, IL, USA), including means, proportions, Odd ratio (OR) with 95% confidence intervals (CIs). Chi square tests were performed to compare proportions of independent variables and t-test was performed to compare means. Cochran-Armitage test for trend was performed to analyze of the trends of multiple birth rates and fetal/neonatal/infant mortality, during the study period. OR with 95% CI was used to test mortality rates between singletons and twins, between singletons and triplets, or between twin and triplets, respectively. Logistic regression analysis was performed to estimate the impact of gestational age on the mortalities among singleton, twin, and triplet births. Because gestational age and mortality demonstrated statistically significant interaction (p< 0.001), GPs were divided into three. Statistical significance was reached with a *P* value of < .05 or if the 95% CIs did not include 1.

## Results

### The numbers of singleton births and multiple births (total, twins, and triplets) and rates

**Fig 1:**
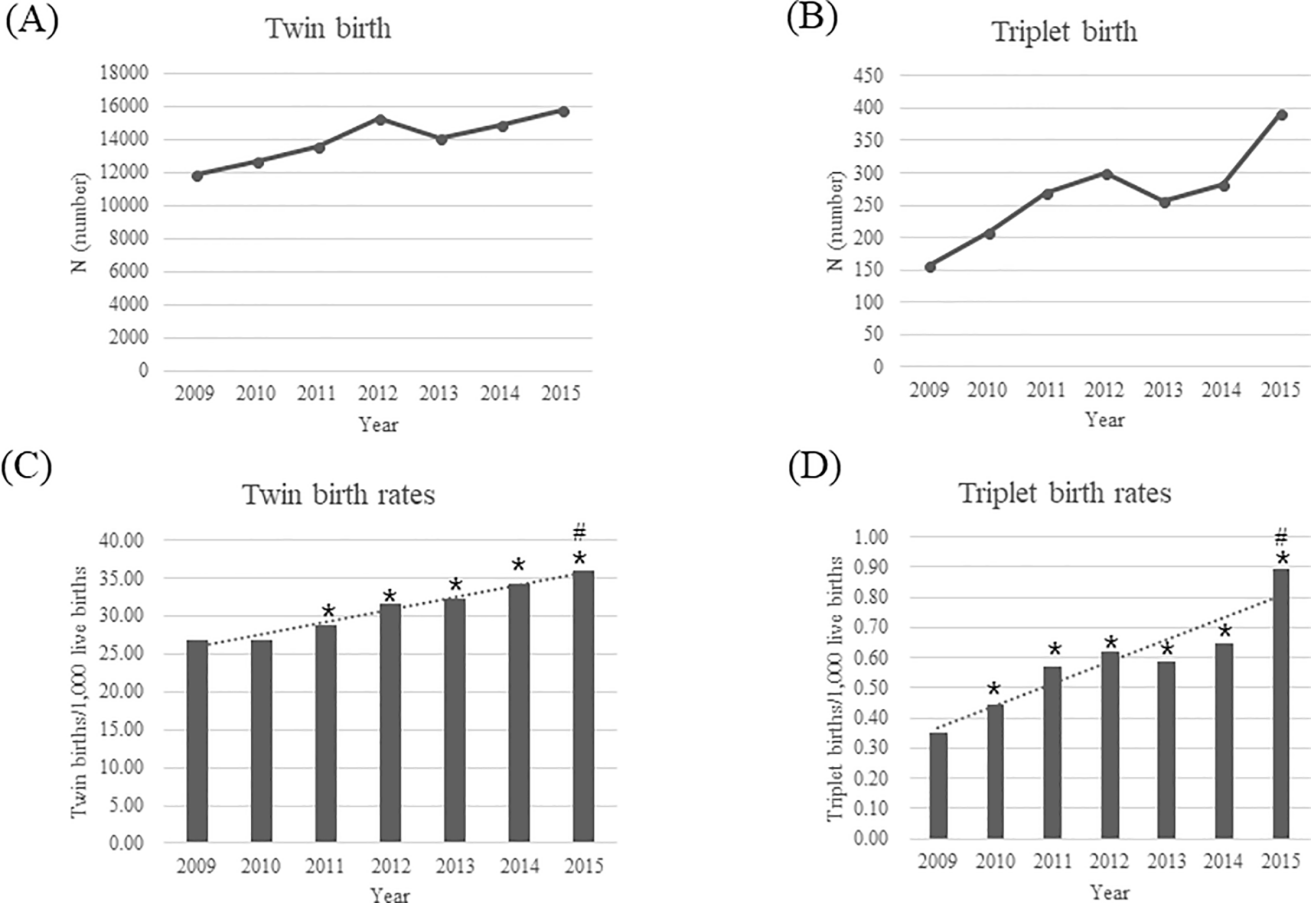
The number of twin and triplet live births and birth rates from 2009 to 2015. (A) Twin births, (B) Triplet births, (C) Twin birth rates, (D) Triplet birth rates.

The numbers of total live births, singleton and multiple births (total, twin, and triplet), and the changes in the MBR, twin births rate, and triplet births rate during 7 years are shown in Table 1. The total live births showed decreasing trends, but the total number of multiple births, and each number of twin and triplet births showed increasing trends (Table 1 and Fig 1). The MBR was steadily increased from 27.11 in 2009, 32.24 in 2012, to 36.87 in 2015. There were 36% (from 27.11 to 36.87), 34.5% (from 26.76 to 35.98), and 154.3% (from 0.35 to 0.89) increases in the MBRs, twin birth rates, and triplet birth rates, respectively, which were statistically significant increasing trends during 7 years (p <0.001).

**Table 1:** Annual changes in number of total live births, multiple births, and multiple birth rate in Korea (2009–2015).

	No. of total	No. of singleton	No. of multiple	No. of twin	No. of triplet	Multiple birth	Twin birth	Triplet birth
Year	live births	births	births^†^	births^†^	births^†^	rates^††^	rates^††^	rates^††^
2009	444849	430983	12062	11905	157	27.11	26.76	0.35
2010	470171	455309	12841	12633	208	27.31	26.87	0.44*
2011	471265	457171	13852	13583	269	29.39*	28.82*	0.57*
2012	484550	468608	15621	15321	300	32.24*	31.62*	0.62*
2013	436455	421837	14372	14115	257	32.93*	32.34*	0.59*
2014	435435	420013	15180	14898	282	34.86*	34.21*	0.65*
2015	438420	421988	16166	15774	392	36.87*^#^	35.98*^#^	0.89*^#^

* p < 0.05, compared with data in 2009.

^#^ p < 0.001, by Cochran-Armitage test for trend, during study period.

†No. of multiple birth: Individual Number of multiples born (not means twin pairs or triplet sets)

^††^Multiple, twin, or triplet birth rate: multiple, twin, or triplet births/1,000 live births.

### Baseline characteristics and NMR, IMR, and FMR in total cohort

The total number of live singleton births and multiple births were 3,075,909 and 100,094, after excluding 5142 births according to the criteria, among total 3,181,445 live births, during 7 years (Table 2). Gestational weeks, birth weight, proportion of preterm births, NMR, IMR, and FMR were described in Table 2. Mean gestational age at birth and birth weight were 38.75 ± 1.53 weeks and 3.24 ± 0.44kg in singleton, 35.59 ± 2.4 weeks and 2.38 ± 0.49 kg in twin, and 32.68 ± 2.94 weeks and 1.75 ± 0.5 kg in triplets, respectively, which were significantly different between singleton and twin, singleton and triplet and between twin and triplet births (all p < 0.001). There were also significant differences in NMRs, IMRs, and FMRs in singleton, twin, and triplet births (all p < 0.001).

**Table 2:** Baseline characteristics according to the number of gestation.

		Singleton births	Twin births	Triplet births	
Live births		n = 3075909	n = 98229	n = 1865	p-value
Gestational weeks (mean ±SD)		38.75 ± 1.53	35.59 ± 2.4	32.68 ± 2.94	< 0.001*^†^
Birth weight (mean ±SD)		3.24 ± 0.44	2.38 ± 0.49	1.75 ± 0.5	< 0.001*^†^
Preterm births at < 24 weeks, n (%)		605 (0.02)	283 (0.29)	27 (1.45)	< 0.001*^†^
Preterm births at ≥24, < 28 weeks, n (%)		4611 (0.15)	1627 (1.66)	83 (4.45)	< 0.001*^†^
Preterm births at ≥28, < 32 weeks, n (%)		16376 (0.53)	5610 (5.71)	480 (25.74)	< 0.001*^†^
Preterm births at ≥32, < 37 weeks, n (%)		142838 (4.64)	53369 (54.33)	1784 (95.66)	< 0.001*^†^

NMR, neonatal mortality rate; IMR, infant mortality rate; FMR, fetal mortality rate; SD, standard deviation.

* statistics between singleton and twin births.

†statistics between twin and triplet births.

### Neonatal mortality, Infant mortality, and fetal mortality trends in singleton, twin and triplet births

**Fig 2:**
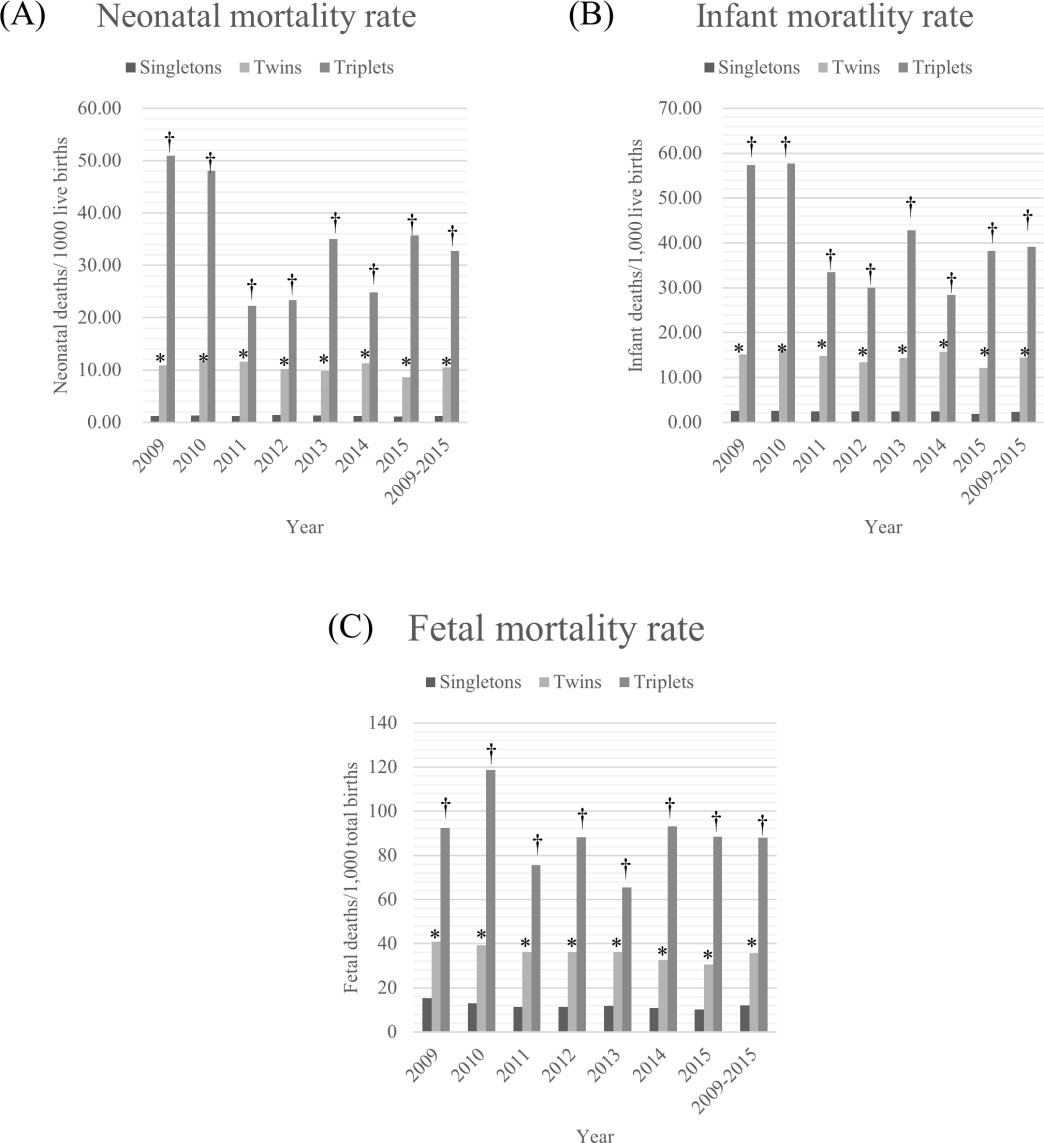
Neonatal mortality rates (A), Infant mortality rates (B), and fetal mortality rates (C) in singleton, twin and triplet births, from 2009 to 2015.

In the period 2009–2015, NMRs have not been decreased significantly in singleton births (from 1.23 to 1.10, p = 0.218) and triplet births (from 50.96 to 35.71, p = 0.402), but NMRs have been significantly decreased in twin births (from 10.92 to 8.62, p = 0.034) (Fig 2 & S1 Table). However, significantly decreasing trend of IMRs were only observed in singletons (from 2.58 to 1.82, p< 0.001). In 2015, the odds ratios of NMRs and IMRs were still 9.07-fold and 6.06-fold higher in twin births, and 37.31-fold and 19.91-fold higher in triplet births, than those in singletons. FMRs were significantly decreased in singletons (from 15.40 to 10.11, p< 0.001) and twins (from 41.00 to 30.55, p< 0.001) (Fig 2 & S2 Table). However, FMRs in triplet births did not show significantly decreasing trend. In 2015, the odds ratios of FMRs in twin and triplet births were 3.17- and 9.55-fold higher than it in singleton births.

### The distribution of gestational age, and NMR, IMR and FMR, stratified by GP, in singleton, twin, and triplet births

Preterm births rates at GP: 24–27, GP: 28–31, GP: ≥32 and < 37 weeks, were significantly different between singleton and twin, singleton and triplet, and between twin and triplet births, which were higher in twin and triplet births (all p < 0.001) (Table 2).

**Fig 3:**
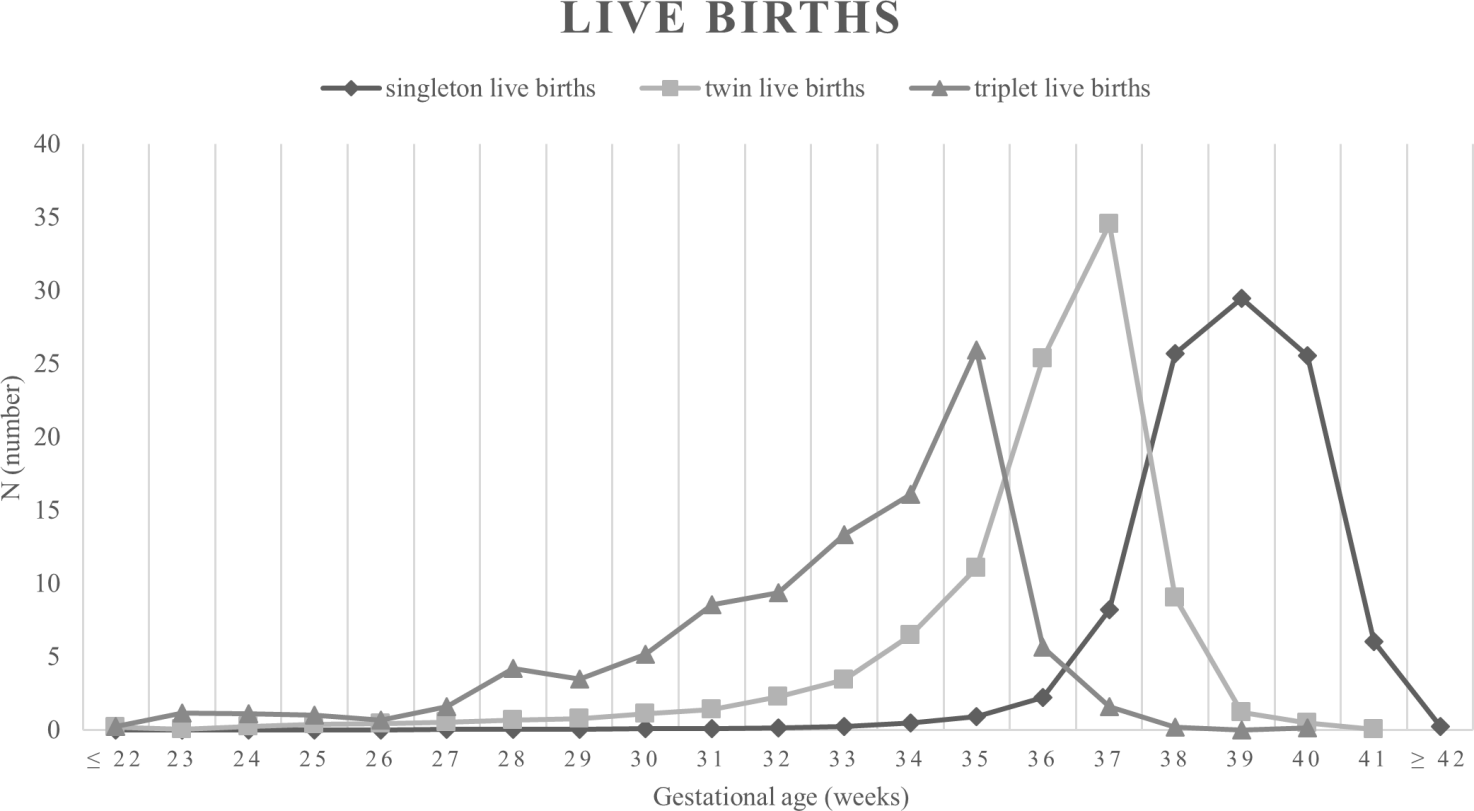
The overall distributions of singleton, twin and triplet births by gestational age.

The Fig 3 shows the overall distributions of singleton, twin and triplet births by gestational age. A shift to the left in the gestational age distribution was evident in twin and triplet births relative to the singleton births, as well as in triplet births relative to the twin births, suggesting higher occurrence of preterm births in twin and triplet births.

The ORs of increasing gestational age by logistic regression analysis on NMR were 0.63 (95% CI, 0.62–0.63) in singleton births, 0.57 (95% CI, 0.56–0.59) in twin births, and 0.56 (95% CI, 0.49–0.63) in triplet births. The ORs of increasing gestational age by logistic regression analysis on IMR were 0.64 (95% CI, 0.63–0.64) in singleton births, 0.59 (95% CI, 0.58–0.60) in twin births, and 0.55 (95% CI, 0.49–0.62) in triplet births. Because there are significant interactions between gestational week and mortalities, we divided gestational age to three groups. When gestational age is subdivided to 3 GPs (GP 24–27, GP 28–31, and GP 32–36 weeks) after 23 gestational weeks, NMRs and IMRs in twin births were significantly higher than those in singleton births, in GP 24–27 and GP 28–31 weeks (Table 3). NMRs and IMRs in triplet births were significantly higher than those in singleton births, in GP 24–27 weeks, but there were no significant differences in in other periods. When gestational age is subdivided to 4 GPs (GP ≤ 23, GP 24–27, GP 28–31, and GP 32–36 weeks), FMRs in twin pregnancy were significantly lower than it in singleton pregnancy, in GP ≤23 and GP 24–27 weeks, but significantly higher than those in singleton pregnancy, in other periods (Table 4). FMRs in triplet pregnancy were significantly lower until 31 weeks compared with those in twin and singleton pregnancies, but 3.4-fold and 2.55- fold higher in GP 32–36 weeks, than those in singleton and twin pregnancies, respectively.

**Table 3:** Neonatal and infant mortality rates, stratified by gestational period.

			Neonatal deaths			
Gestational period	Singletons	Twins	Odds ratio*	Triplets	Odds ratio**	Odds ratio^†^
(weeks)	n (NMR)	n (NMR)	95% CI	n (NMR)	95% CI	95% CI
24–27	1090 (236.39)	440 (585.89)	**4.58 (3.78–5.56)**	24 (489.8)	**3.11 (2.57–3.77)**	**0.68 (0.57–0.81)**
28–31	506 (43.01)	170 (100.24)	**2.47 (1.71–3.58)**	10 (57.14)	1.35 (0.9–2.12)	**0.54 (0.39–0.76)**
32–36	602 (4.76)	124 (6.02)	1.2 (0.37–3.95)	3 (5.32)	1 (0.29–3.47)	0.83 (0.25–2.74)
			Infant deaths			
Gestational period	Singletons	Twins	Odds ratio*	Triplets	Odds ratio**	Odds ratio^†^
(weeks)	n (IMR)	n (IMR)	95% CI	n (IMR)	95% CI	95% CI
24–27	1517 (329)	575 (585.89)	**6.68 (5.48–8.13)**	25 (510.2)	**2.12 (1.77–2.54)**	**0.32 (0.26–0.39)**
28–31	758 (64.43)	235 (138.56)	**2.36 (1.73–3.22)**	11 (62.86)	0.98 (0.69–1.41)	**0.42 (0.30–0.57)**
32–36	1043 (8.25)	222 (10.78)	1.38 (0.55–3.44)	2 (3.55)	0.5 (0.15–1.66)	0.36 (0.11–1.14)

NMR, neonatal mortality rate; IMR, infant mortality rate; CI, confidence interval

* statistics between singleton and twin births.

** statistics between singleton and triplet births.

†statistics between twin and triplet births.

**Table 4:** Fetal mortality rates stratified by gestational period.

			Fetal deaths			
Gestational period	Singletons	Twins	Odds ratio*	Triplets	Odds ratio**	Odds ratio^†^
(weeks)	n (FMR)	n (FMR)	95% CI	n (FMR)	95% CI	95% CI
< 24	27899 (978.77)	2460 (947.61)	**0.39 (0.23–0.65)**	137 (913.33)	**0.23 (0.14–0.37)**	**0.58 (0.40–0.82)**
24–27	4430 (489.99)	338 (310.38)	**0.47 (0.39–0.56)**	10 (169.49)	**0.21 (0.17–0.26)**	**0.45 (0.37–0.56)**
28–31	1773 (130.96)	239 (123.51)	0.94 (0.72–1.22)	12 (64.17)	**0.45 (0.33–0.62)**	**0.48 (0.35–0.66)**
32–36	1756 (13.70)	452 (21.48)	1.51 (0.76–2.99)	27 (45.69)	**3.4 (1.85–6.22)**	**2.25 (1.33–3.80)**

FMR, fetal mortality rate; CI, confidence interval

* statistics between singleton and twin births.

** statistics between singleton and triplet births.

†statistics between twin and triplet births.

## Discussion

In the period 2009–2015, MBR increased 36.4%, twin birth rate increased 34.5%, triplet birth rate increased 154.3%. It was reported that MBR increased from 10.0 to 20.0 between 1991 and 2003, 20.0 to 27.5 between 2004 and 2008, in Korea [8]. The current study shows that the incidence of multiple births in Korea has been increased, and the increase of triplet births has been more pronounced, within recent 7 years. In Korea, the proportion of advanced maternal age (≥35 years old) has been increased from 10.5% in 2005 to 23.9% in 2015 [9]. It is well known that advanced maternal age is strongly associated with multiple births, and the parallel increase of multiple births with the use of ART implies the importance of the association of ART with multiple births in Korea [8]. The total fertility rate in Korea was 1.24 persons in 2015, which was the lowest among the OECD nations [9], and the number of total liver births during study period showed decreasing trend. Therefore, it is important to evaluate pregnancy outcomes of multiple pregnancies, with making efforts to increase total fertility rate.

As expected, this study demonstrated that NMRs and IMRs in multiple births were significantly higher than those in singleton births, throughout the study period. Mean gestational age and birth weight were significantly lower in twin and triplet births than those in singleton births. Previously, mean gestational age in triplet births in other studies was reported as approximately 33.5 weeks, approximately 25% deliver at <32 weeks and 10% deliver < 28 weeks [10]. However, those other studies were performed before 2000 year. This study showed that 4.45% of triplet births and 1.66% of twin births were occurred before 28 gestational weeks, compared to 0.15% of singleton births. Thus, preterm births rate (at ≥ 24 and < 28 weeks) in triplets seems lower than those of previous reports, but mean gestational age (32.8 weeks in this study) is also little bit lower. Fetoscopic laser surgery on Twin-to-Twin transfusion syndrome (TTTS) became available after 2010 in Korea and it might be related with small reduction of NMR and IMR [11]. However, physicians should recognize that the odds ratios of NMRs and IMRs are approximately 9-fold and 6-fold higher in twin births, and approximately 37-fold and 20-fold higher in triplet births, than those in singleton births, and counsel the patients who receive ART, to decrease the number of transferring embryos and increase neonatal and infant survival.

When NMRs and IMRs were stratified by GP, NMRs and IMRs in twin births were significantly higher in births before 32 weeks, and NMRs and IMRs in triplet births were significantly higher in births before 28 weeks, after then there were no significant differences between triplet and singleton births. In addition, NMRs and IMRs in triplet births were significantly lower than those in twin births, before 32 weeks.

It is known that babies born following assisted reproductive technology (ART) treatment, as these births have been associated with the higher rates of prematurity, small for gestational age, multiple births and congenital anomalies when compared with spontaneous conceptions [12–14]. The higher NMRs and IMRs in multiple births can be related with ART and related complications, but the result of current study suggests that the main reason is high rate of preterm birth in multiple births, because those became non-significant in later gestation. A recent study also reported that the risk of respiratory morbidity among late-preterm twins was similar to that of late-preterm singletons [15]. Interestingly, survival seems better in triplet births at the same gestation than twin births, before 31 weeks. Although it still remains unknown whether fetal growth restriction or other stressful condition during pregnancy accelerates lung growth or maturation, or make abnormal pulmonary vascular development [16–18], a recent multicenter trial reported that SGA is not associated with RDS or other adverse respiratory and neonatal composites. The logistic regression analysis shows that the risks of NMR and IMR in multiple births at increasing gestational age, decreased further significantly in comparison with singleton births. We speculated that comparable outcomes in NMRs and IMRs, between multiple births and singleton births, at 32–36 weeks, might be related with accelerated lung maturation in multiple births. The possible reason for better survival in triplet births at same GP, compared with survival in twin births, can be more frequent monochorionicity and related complication in twin pregnancy. Similarly, A study from Japan reported that IMRs in twin preterm births were lower than those in singleton preterm births, between 30–36 weeks [19]. A long term Nordic study suggested that ART twins had a lower risk of early neonatal and infant deaths than twins by spontaneous conception [20]. Because other studies reported that multiple gestation itself is related with poorer perinatal outcomes and neurodevelopment, further studies are needed [21, 22].

Because there is no effective prevention or treatment methods related with preterm labor in multiple pregnancy, yet, the strategy for decreasing high-order pregnancies is urgent. Moreover, in Japan, NMR and IMR in twin births were reported as 4.9 and 11.5 per 10,000 live births in 2008, which were lower than 8.62 and 12.5 per 10,000 live births in 2015, respectively, in Korea. It was reported that NMR and IMR in Korea were 1.8 and 3.2 per 1,000 total live births, respectively, significantly lower than in the U.S. of 4.1 and 6.2 per 1,000 live births respectively, but higher than in Japan, 1.1 and 2.1 per 1,000 live births respectively [23], in the period 2009–2010. Thus, antenatal care and the intensive care of multiple births during neonatal period need to be more improved.

Overall FMRs in twin and triplet birth were significantly higher than it in singleton births, during the study period. The higher rates are because of both complications specific to multiple gestations (such as TTTS) and to overall increased risks of complications, such as fetal congenital abnormalities and growth restriction [24]. When FMRs stratified GP, however, were lower than it in singleton births, before 28 weeks, in twin births, and 32 weeks in triplet births. After 27 weeks, there were no significant differences in FMRs between twin and singletons, but after 32 weeks, FMRs in triplets became significantly higher than those in singleton and twin births. The reason for lower FMRs in twin and triplet pregnancies, until 27 weeks and 31 weeks, respectively, is unclear and complicated. However, the FMRs in multiple gestation during the third trimester should be recognized in physician and patients, especially in patients who are considering high-order embryo transfer. In addition, patients should be counseled appropriately regarding the risks and benefits of the multifetal pregnancy reduction procedure, when they conceive high-order gestation, in case of double or multiple embryos transfer, including the fetal/neonatal/infant mortalities and morbidities, as well as risks of miscarriage and long term adverse outcomes. Some advocate multifetal pregnancy reduction to twins from higher-order pregnancies, to increase gestational age and improve maternal and fetal outcomes [11]. However, it was reported that multifetal pregnancy reduction is related with the increase approximately 10% risks of miscarriage [25]. Suggested mechanisms were ‘procedure-related trauma or infection, in which case the miscarriage would be expected within 2 weeks of reduction’, and ‘the consequence of the resorbing dead fetoplacental tissue, which could result in miscarriage several weeks or months after multifetal reduction. It is reported that multifetal reduction is associated with an increase in maternal serum α-fetoprotein concentration that is proportional to the amount of dead fetoplacental tissue, and this increase persists for several months following the procedure [26].

The American Society of Reproductive Medicine and the European Society of Human Reproduction and Embryology set strategies involving reducing the number of embryos transferred per IVF cycle, or attempting a greater number of antiestrogen/intrauterine insemination cycles before the use of gonadotropins in ovulation induction treatments [27].

There has been a decline in perinatal mortality among babies born after ART in Australia and New Zealand from 24.7 per 1000 births following ART treatment in 1999 to 16.2 per 1000 births following ART treatment in 2008, in accordance with the increase in the proportion of SET, from 14.2% in 1999 to 67.8% in 2008 and the accompanying 13.7% reduction in the MBR from 22.1% in 2000 to 8.4% in 2008 [3, 28].

The recent study from European countries which have different MBRs proved that the countries with lower MBR have lower risk of preterm birth, as well as lower fetal and neonatal mortalities [6]. Among the countries, the highest MBR country was Cyprus with 26.5 of MBR, in 2010. In this study, MBR in 2010 showed 27.3 in Korea, which is higher than it of Cyprus in 2010. Moreover, it has shown increasing trend until 2015, especially triplet birth rates.

The limitation of this study is mainly from the deficient details of multiple pregnancies, such as chorionicity, use of ART, details of ART including information related with multifetal reduction, and so on. However, this study provides fetal, neonatal, and infant mortalities of twin and triplet pregnancies and recent trends of MBR, as well as twin and triplet birth rates, in a population level. The other limitation is that we did not assess the effects of possible influencing factors such as number of siblings (singleton, twin or others), fetal/neonatal gender and mother's age and so on, due to the data limitation.

Low birth rates and pregnancies in women at ≥ 35 are national problems in Korea. ART contributes increasing birth rates. However, increase of high-order gestation can cause not only perinatal adverse outcome, but also long -term social and health problem, mainly from preterm births, in a national level. The obstetric and gynecologic doctors related with ART and antenatal care, neonatologists, pediatrician, as well as politicians and patients, should make efforts to decrease MBR, enabling better short-term and long-term child health, with improving antenatal and postnatal care, especially related with preterm births in multiple pregnancies, and to increase overall birth rates. In addition, close fetal monitoring is needed to prevent fetal death in triplet pregnancies, after 32 gestational weeks.

## Supporting information

S1 Table Annual neonatal and infant mortalities and comparisons of mortality rates according to the number of gestation. (XLSX)

S2 Table Annual fetal mortalities and comparisons of fetal mortality rates according to the number of gestation. (XLSX)
